# Digital Media’s Role in the COVID-19 Pandemic

**DOI:** 10.2196/20156

**Published:** 2020-09-18

**Authors:** Huanyu Bao, Bolin Cao, Yuan Xiong, Weiming Tang

**Affiliations:** 1 UNC Project-China Guangzhou China; 2 Shenzhen University Shenzhen China

**Keywords:** COVID-19, digital health, media, pandemic, public health, social media, dissemination, health information, mobile health

## Abstract

The severe acute respiratory syndrome coronavirus 2 outbreak has had a significant impact on global health, the economy, and society as a whole. Various measures are being taken to respond to the pandemic, with digital media playing a pivotal role, especially in the use of visual data to disseminate information, mobile health to coordinate medical resources, social media to promote public health campaigns, and digital tools to assist population management and disease tracing. However, digital media also faces some challenges like misinformation, lack of guidance, and information leakage. We encourage the increased use of digital media with a focus on improving trust, building social solidarity, reducing chaos, educating the public on prevention measures, and reducing the medical burden in facility-based sites.

As of May 31, 2020, the coronavirus disease (COVID-19) outbreak has led to the death of 367,166 people [1]. The pandemic is causing severe damage to the health care system, the economy, and society as a whole. Plans and actions to prevent and respond to the pandemic are urgently needed. Of these, the digital media’s response, advocate, and mobilization plays an essential role. With the development of information and technology, digital media plays a pivotal role in this pandemic, especially in the use of visual data to disseminate information, mobile health (mHealth) to coordinate medical resources, and social media to promote public health campaigns.

First, visual data is used increasingly to demonstrate the distribution, transmission, and trend of this coronavirus outbreak. The unprecedented pandemic has brought an enormous amount of real time data, and many online media platforms adopted visual graphs to release COVID-19 statistics, which were rarely used during the severe acute respiratory syndrome outbreak. Data visualization can help people easily and efficiently process a large volume of information on disease transmission to understand the patterns of epidemics [2]. An example of this is the interactive dashboard developed by Johns Hopkins University based on the crowdsourcing data [3]. This dashboard provides data-driven visuals (eg, global cases map, critical data trends, latest news, and COVID-19 basics) to illustrate the situations of pandemics around the world, enabling the public and researchers to understand and monitor the outbreak timely. Similarly, a popular messaging app (WeChat) in China offered a location-based feature, “Cases Nearby,” to show the location of the confirmed cases around the users and the places the cases have been without disclosing any personal information [4]. This visual footprint keeps users informed of the outbreak and advises them to take targeted measures to avoid high-risk areas. In addition, in the Prince of Wales Hospital of Hong Kong, an infographic on the principles of airway management was developed in 17 languages and disseminated through online social network platforms, benefiting other medical units to incorporate infection control procedures to reduce the transmission of COVID-19 [5].

Second, mHealth is surging in demand to reduce the overloading of health care systems. To avoid the high risk of contact with infected individuals, several virtual teleconsultation platforms (*EmergencyEye* in Germany, *Vodacom* in South Africa, and *WeDoctor* in China) were used to assist health care professionals. Facebook groups have been used by doctors to share and integrate experiences in disease treatment and research in real time, a subgroup called the PMG COVID19 has 36,900 members worldwide [6]. The pandemic has also driven research and the application of artificial intelligence (AI) in dealing with this emerging issue. By using lung computed tomography scans, AI technology was used to help doctors make a quick judgment of coronavirus pneumonia [7]. To help fight mental health disorders during the pandemic [8], an AI-based chatbot has also played important roles in responding to people’s emotions and providing online consultation. This trend was witnessed by the surge in users for some Indian chatbot software during the outbreak [9].

Third, social media platforms are applied to educate people to take public health measures. As one of the first countries hit by COVID-19, Singapore’s successful response has benefited from the early action taken by the country via social media. A national WhatsApp channel was immediately created to inform people living in Singapore about government updates and initiatives on COVID-19. There have been over 635,000 people subscribed to the channel to receive updated messages [10]. In China, the government has partnered with mobile phone operators to send automated text messages at various times throughout the day to keep people informed and alert them to keep a social distance. Additionally, in partnership with the health ministry, a Vietnamese music artist wrote a song, and a local dancer choreographed a dance on how to wash hands carefully and started a dance challenge on TikTok (a popular video-sharing app) [11]. The dance challenge video has gone viral and invited millions of people to learn about the essential steps of handwashing, playing a critical role in fighting against the spread of COVID-19.

Fourth, digital tools are applied to assist the management of work resumption and citizen migration after the pandemic. In China, the digital health code, which displays a Quick Response (QR) code with an individual’s health status, is widely used to track citizen’s health status and estimate their potential risk in transmitting the virus. Individuals are assigned a color code—green, yellow, or red—that indicates their health status. The functions of digital health codes are two-fold: for ensuring anyone entering a public place is healthy and for contact tracing purposes. Although such digital tools have raised concerns about privacy, it helps to contain the outbreak of epidemics and mitigate the burden of public health surveillance, allowing society to return to normal. In addition, coronavirus tracking apps were also applied with the official government in other countries to a id contact tracing, such as Australia (*COVIDSafe*), Bahrain (*BeAware Bahrain*), Colombia (*CoronApp*), and Ghana (*GH Covid-19 Tracker App*).

Although digital media has made considerable efforts in response to the pandemic, it is still facing some challenges. First, misinformation is a pressing problem. Rumors, fake news, and deliberate misinformation have been spreading on social media platforms, causing distrust and further endangering public health [12,13]. To respond to the infodemic caused by misinformation, some efforts have been made to correct the misinformation. Useful corrective actions such as more coherent information that provides alternative explanations to misleading information and appeals to credibility should be continuously, widely, and frequently distributed [14]. Second, there is a lack of formalized guidance to guide the use of digital media in large-scale epidemics. In particular, personal privacy and data leakage is an issue that needs to be addressed urgently. *Zoom*, the teleconferencing software that was heavily used in this outbreak, suffered several hacks [15], which raised concerns about the security of digital tools. Although the digital health code has been used in several countries, there is still a concern about privacy, and personal information leakage may cause geographical discrimination against people from high-risk areas.

The COVID-19 pandemic is continuing to worsen, and more effective strategies are needed. Although digital media has played an important role, we strongly recommend that it should be further used to improve trust, build social solidarity, reduce chaos, educate the public for prevention measures, and reduce the medical burden in facility-based sites. Only by using multiple resources and working together globally, can we mitigate the effects of COVID-19, even if this comes at a cost.

## Abbreviations

AI: artificial intelligence
COVID-19: coronavirus disease
mHealth: mobile health
